# One-year outcomes and safety assessment of faricimab in treatment-naïve patients with neovascular age-related macular degeneration in Japan

**DOI:** 10.1038/s41598-024-62559-1

**Published:** 2024-05-22

**Authors:** Ryo Mukai, Keiko Kataoka, Koji Tanaka, Yasunori Miyara, Ichiro Maruko, Makiko Nakayama, Yuto Watanabe, Akiko Yamamoto, Yu Wakatsuki, Hajime Onoe, Sorako Wakugawa, Nobuhiro Terao, Taiji Hasegawa, Moeko Kawai, Ruka Maruko, Kanako Itagaki, Jyunichiro Honjo, Annabelle A. Okada, Ryusaburo Mori, Hideki Koizumi, Tomohiro Iida, Tetsuju Sekiryu

**Affiliations:** 1 Department of Ophthalmology, Fukushima Medical University, 1 Hikarigaoka-cho, Fukushima, 960-1295 Japan; 2 Department of Ophthalmology, Kyorin University School of Medicine, Tokyo, Japan; 3 Department of Ophthalmology, Nihon University School of Medicine, Tokyo, Japan; 4 Department of Ophthalmology, Graduate School of Medicine, University of the Ryukyus, Okinawa, Japan; 5 Department of Ophthalmology, Tokyo Women’s Medical University, Tokyo, Japan

**Keywords:** Faricimab, Neovascular age-related macular degeneration, Treat-and-extend regimen, Visual acuity, Retinal morphology, Choroidal thickness, Eye diseases, Outcomes research

## Abstract

This multicentre retrospective study evaluated the 1-year outcomes and safety profile of faricimab in treatment-naïve patients with neovascular age-related macular degeneration (nAMD). Fifty-five patients (57 eyes) underwent loading therapy comprising three monthly faricimab injections. If dryness was achieved by the third month, subsequent treat-and-extend (TAE) follow-up continued at a minimum 8-week interval thereafter. If wet macula persisted at the third month, a fourth dose was administered, followed by the TAE regimen. After 1 year, improvements in visual acuity (0.44 ± 0.46 [baseline] to 0.34 ± 0.48; p < 0.01) and central foveal thickness (326 ± 149 [baseline] to 195 ± 82 μm; p < 0.0001) were significant. Dry macula, characterised by the absence of intraretinal or subretinal fluid, was achieved in 65% of cases. Treatment intervals varied, ranging from 8 to 16 weeks, with 44% of eyes extending to a 16-week interval, followed by 33% at 8 weeks, 16% at 12 weeks, 5% at 14 weeks, and 2% at 10 weeks. Notably, 50% of the polypoidal choroidal vasculopathy patients exhibited complete regression of polypoidal lesions between 12 and 15 months. Faricimab treatment in nAMD patients induced significant improvements in central vision and retinal morphology. Two cases of retinal pigment epithelial tears and one case of iritis were reported as ocular complications.

## Introduction

Faricimab, a novel bispecific antibody, has emerged as a promising agent against macular neovascularisation (MNV) in patients with neovascular age-related macular degeneration (nAMD). Its ability to bind to both vascular endothelial growth factor-A (VEGF-A) and angiopoietin-2 (Ang-2), key players in the development of MNV, makes it a unique therapeutic approach^1–4^. The TENAYA (NCT03823287) and LUCERNE (NCT03823300) international phase III clinical trials investigated the efficacy of faricimab in patients with nAMD. Over the span of 2 years, these trials demonstrated significant enhancements in both visual acuity and retinal morphology^5^. Following approval from the Japanese governmental, faricimab became globally available from 2022 onwards.

Our previous study detailed the short-term outcomes of faricimab in the treatment of nAMD and revealed that a loading therapy regimen substantially improved both visual acuity and retinal morphology in treatment-naïve patients without any particular safety issues^6^. In addition, a dry macula was achieved in 82% of the eyes after the loading therapy. Further, complete regression of polypoidal lesions was observed in 52% of eyes with polypoidal choroidal vasculopathy (PCV).

The TENAYA and LUCERNE trials demonstrated a sustained improvement in visual acuity following loading doses, consistently observed in both the faricimab and aflibercept treatment groups over a period of 1 year. In the faricimab group, treatment intervals were adjusted to 8, 12, or 16 weeks based on disease activity at 20 or 24 weeks. Notably, by the 48-week mark, approximately 78–80% of patients were allocated a treatment interval of either 12 or 16 weeks^5^. However, real-world clinical settings often employ the treat-and-extend (TAE) regimen^7^ as a common treatment strategy. Despite its widespread use, there is currently a dearth of reports on the treatment outcomes of faricimab when utilising TAE regimens over a period of 1 year. This knowledge gap underscores the need for a comprehensive investigation of the real-world effectiveness of faricimab in the context of TAE protocols. Such an exploration would provide valuable insights into its performance and potential advantages in everyday clinical practice.

This report aimed to present the comprehensive 1-year treatment outcomes of faricimab for nAMD using a TAE regime, with a focus on visual acuity, retinal morphology, choroidal thickness, and other pertinent clinical profiles.

## Methods

### Study and institution

This multicentre, retrospective, observational study was conducted in accordance with the Tenets of the Declaration of Helsinki and was granted approval by the Institutional Review Boards of Kyorin University, Fukushima Medical University, Tokyo Women’s Medical University, University of Ryukyus, and Nihon University (Japan AMD Research Consortium: JARC). Given the retrospective nature of the study, each Institutional Review Board waived the need for individual patient informed consent. Details of the study were shown on the respective homepages of each university hospital, allowing patients the opportunity to opt out of participation if they chose to do so.

### Participants

This study included 70 treatment-naive eyes of 68 patients with nAMD who received their first injection of faricimab between June 2022 and October 2022. All patient data were anonymised prior to analysis. The included patients were treatment-naive and aged ≥ 45 years. All patients underwent ophthalmologic examinations, fluorescein angiography (FA), and indocyanine green angiography (ICGA) using a confocal scanning laser ophthalmoscope (Spectralis HRA + optical coherence tomography [OCT]; Heidelberg Engineering, Germany) to determine the subtypes of nAMD, including type 1 MNV, type 2 MNV^8^, PCV^9^, and type 3 MNV^8^. PCV was diagnosed based on ICGA-visualized polypoidal lesions^9^. The diagnostic criteria for type 3 MNV included the presence of retinal–retinal anastomosis on early-phase FA or ICGA and a hotspot on late-phase ICGA^10^. Patients with myopia exceeding -6 dioptres, a history of uveitis or vitrectomy, and those with a massive submacular haemorrhage extending beyond the equator for PCV were excluded. Thirteen patients were excluded for the following reasons: six did not return due to personal reasons, five switched to aflibercept, one passed away, and one developed iritis. Therefore, we included 57 eyes of 55 patients.

### Procedures

**Figure 1:**
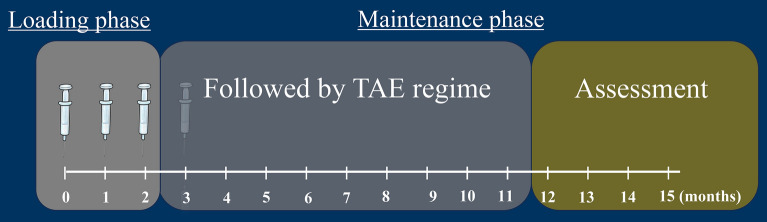
Treatment schedule for optimising neovascular age-related macular degeneration management: combining loading dose therapy with a treat-and-extend (TAE) regimen. Patients who achieved macular dryness by the third month were followed-up with a TAE regimen, with intervals ranging from a minimum of 8 weeks to a maximum of 16 weeks. Patients with persistence of wet macula at the third month were administered a fourth dose, followed by the TAE regimen.

Intravitreal faricimab (IVFa) injections were administered to all eyes (6 mg/0.05 mL). During loading therapy, the patients received three monthly injections of faricimab. Patients who achieved dryness by the third month were followed-up with the TAE regimen. For patients whose wet macula persisted by the third month, a fourth dose was administered, followed by the TAE regimen. During the maintenance phase, the interval between injections was adjusted based on the presence or absence of exudative changes: If no exudative changes were detected, the interval was extended by 2–4 weeks. However, if exudative changes were detected, the interval was shortened by 2–4 weeks. The treatment was continued for a minimum interval of 8 weeks and a maximum interval of 16 weeks. The treatment schedule is illustrated in Fig. 1.

Patients who received the minimum number of IVFa injections, i.e. those who did not show any exudative changes during the maintenance phase, visited our hospital at 40 weeks and then again at 60 weeks. To assess these patients at 1 year, it was necessary to follow-up all patients until 60 weeks (15 months). Throughout the course of the study, specifically at the 12th, 13th, 14th, and 15th month, we assessed outcomes including visual acuity, fluid resolution rate, central foveal thickness (CFT), subfoveal choroidal thickness (SCT), changes in pigment epithelial detachment (PED), and polypoidal lesion regression rate in patients with PCV. At Nihon University, we determined the best corrected visual acuity (BCVA) using the early treatment diabetic retinopathy study visual acuity chart, while at the other four institutions, we used the Landolt C chart for the same purpose. BCVA was converted to the logarithm of the minimal angle of resolution (logMAR) units for outcome analyses. The macula was considered dry in cases of complete resolution of the subretinal and intraretinal fluids on OCT images. CFT was measured from the superior border of the retinal pigment epithelium (RPE) to the border of the inner retinal layer at the foveal centre. Additionally, SCT was measured as the vertical distance between the hyperreflective line corresponding to the Bruch’s membrane under the RPE and the inner scleral boundary at the foveal centre. This measurement was conducted using the calliper function of OCT (DRI-OCT [Topcon]) at the University of Ryukyus and Tokyo Women’s Medical University and the Heidelberg Spectralis (Heidelberg Engineering Inc., Germany) at Nihon University, Fukushima Medical University, and Kyorin University.

In the OCT examination, we obtained B-mode images of the horizontal and vertical line scans (12 mm or 30°) through the fovea employing the OCT machines. PED, characterised by one or more detached areas in the macula on FA similar in size to the optic disc, was assessed in this study. Its height was then recorded for comparison with previous measurements. The height of the PED from the inner layer of the Bruch’s membrane to the top of the RPE was measured using OCT. Changes in polypoidal lesions were recorded as complete regression, partial regression (reduction in the number of polypoidal lesions), or an increase relative to baseline levels as measured using ICGA. The presence of subretinal fluid, intraretinal fluid, or sub-RPE fluid was evaluated using volumetric scan OCT (9 × 12-mm volume scan for DRI-OCT or 20° volume scan for Heidelberg-OCT) images covering the entire macula.

With regard to the individualised patient follow-up intervals implemented within the TAE regimen, we employed the last observation carried forward (LOCF) method to manage missing values in monthly data points, following past data patterns. Missing data were also implicitly imputed using a mixed model for repeated measures (MMRM). Where necessary, the reliability of the results of the analysis was evaluated by employing either observed case data or data from completers. Completers were defined as patients who underwent treatment for a minimum of 12 months.

### Statistical analysis

Data are presented as the mean ± standard deviation (SD). The Wilcoxon signed-rank test was used to assess the changes in visual acuity. A one-way analysis of variance was used to compare the CFT and SCT before and after treatment. Statistical significance was set at *p* < 0.05. GraphPad Prism version 10 (GraphPad Software, LLC, USA) was used for the statistical analyses.

## Results

**Figure 2:**
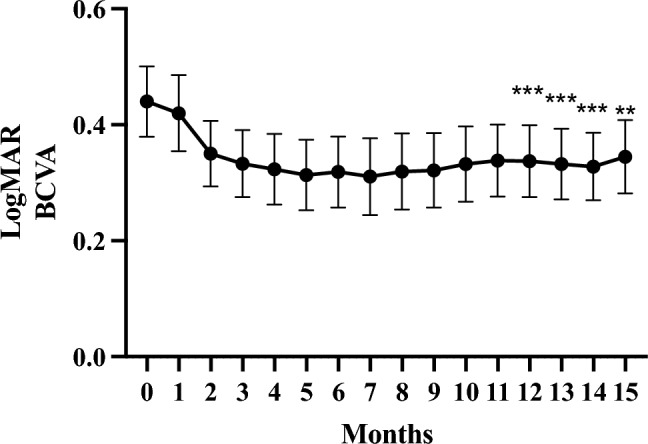
Changes in best-corrected visual acuity (BCVA) in 57 eyes treated with faricimab for 1 year. The data were analysed using the last observation carried forward (LOCF) and shown as the mean ± standard error. **p < 0.01, ***p < 0.001.

**Figure 3:**
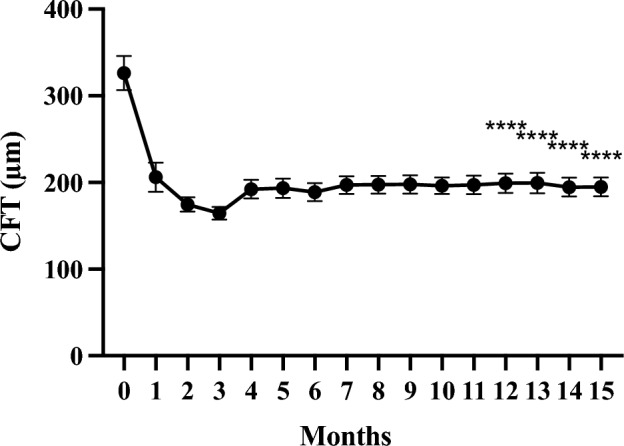
Changes in average central foveal thickness (CFT) in 57 eyes treated with faricimab injections for 1 year. The data were analysed using the last observation carried forward (LOCF) method and shown as the mean ± standard error. ****p < 0.0001.

**Figure 4:**
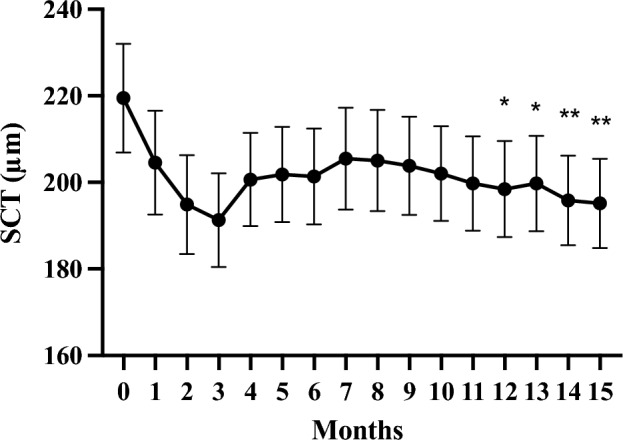
Changes in average subfoveal choroidal thickness (SCT) in 57 eyes treated with faricimab injections for 1 year. *p < 0.05, **p < 0.01.

Table 1 displays the demographic and clinical characteristics of the enrolled patients. Out of the 57 eyes included, 30 (53%) were diagnosed with type 1 and/or type 2 MNV (20 eyes with type 1 MNV, five eyes with type 2 MNV, and five eyes with both), 20 (35%) had polypoidal choroidal vasculopathy (PCV), and seven (12%) exhibited type 3 MNV. The final follow-up assessments were conducted at varying intervals: five eyes were examined at the 12-month mark, nine eyes at 13 months, 26 eyes at 14 months, and 17 eyes at 15 months. The mean BCVA in logMAR at baseline and 1 year (the mean BCVA in logMAR at the final visit within the 12–15-month period) were 0.44 ± 0.46 and 0.34 ± 0.48 respectively, demonstrating significant improvement (p < 0.01). In addition, the mean BCVA in logMAR at 12, 13, 14, and 15 months after initiation of faricimab using LOCF was 0.34 ± 0.47, 0.33 ± 0.46, 0.33 ± 0.44, and 0.34 ± 0.48, respectively. The BCVA exhibited a significant improvement from 12 to 15 months compared to baseline (*p* < 0.001, *p* < 0.001,* p* < 0.001, and *p* < 0.01, respectively; Fig. 2). The mean CFT values at baseline and at 12, 13, 14, and 15 months after the initiation of faricimab were 326 ± 149 μm, 199 ± 84 μm, 199 ± 88 μm, 195 ± 82 μm, and 195 ± 82 μm, respectively. The CFT was significantly reduced at all post-treatment time points (*p* < 0.0001), as shown in Fig. 3. The mean SCT values at baseline and at 12, 13, 14, and 15 months after initiation of faricimab were 220 ± 95 μm, 199 ± 84 μm, 200 ± 84 μm, 196 ± 78 μm, and 195 ± 78 μm, respectively. SCT was significantly reduced at all post-treatment time points (*p* < 0.05, *p* < 0.05,* p* < 0.01, and *p* < 0.01, respectively), as shown in Fig. 4.

**Table 1:** Baseline demographic and clinical characteristics of patients with neovascular age-related macular degeneration.

	All participants
Number of patients (eyes)	55 (57)
Female, n (%)	15 (27%)
Age (years ± SD)	76.1 ± 9.9
Subtype, eyes (%)	
Type 1 and/or type 2 MNV	30 (53%)
Type 1 MNV	20
Type 2 MNV	5
Mixture of type 1 and type 2 MNV	5
PCV	20 (35%)
Type 3 MNV	7 (12%)

*PCV* polypoidal choroidal vasculopathy.

**Figure 5:**
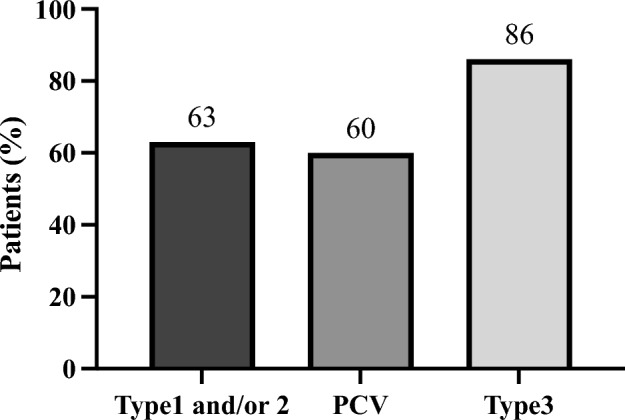
Total number of faricimab injections over 1 year.

The changes in BCVA, CFT, and SCT were also analysed using MMRM, as illustrated in Supplementary Table S1 online. The dry macula achievement rates were as follows: 37/57 eyes (65%) at 1 year, 19/30 eyes (63%) in type 1 and/or type 2 MNV, 12/20 eyes (60%) in PCV, and 6/7 eyes (86%) in type 3 MNV (Fig. 5). Polypoidal lesions in 10 patients with PCV were evaluated using ICGA at 1 year. Our results indicated no detection, partly remaining, and recurrence in five (50%), four (40%), and one (10%) eye, respectively. In the remaining patients with PCV, ICGA was not performed at 1 year because of a lack of consensus. In PCV, a pre-treatment assessment revealed the presence of SRF in 19 cases and IRF in three cases. After a one-year follow-up, SRF was detected in eight cases, while IRF was not detected in any case. Notably, a dry macula was achieved in 12 out of 20 cases, representing a 60% success rate. During the pre-treatment evaluation, PED was identified in 11 cases. Subsequent assessments demonstrated varied outcomes for PED, with exacerbation observed in two cases, while improvement or complete resolution occurred in four cases during the last visits.

**Figure 6:**
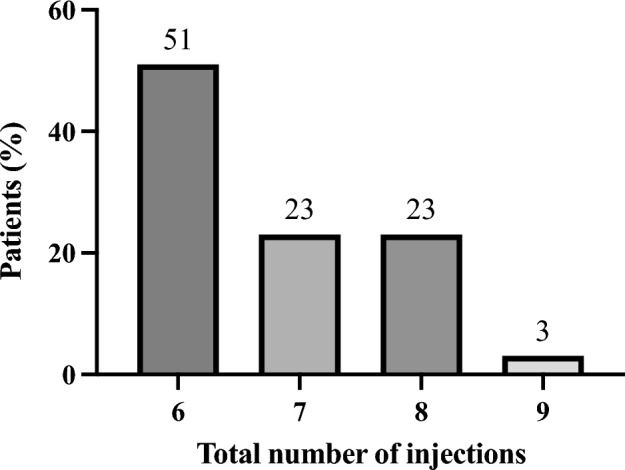
The treatment interval for 1 year.

**Figure 7:**
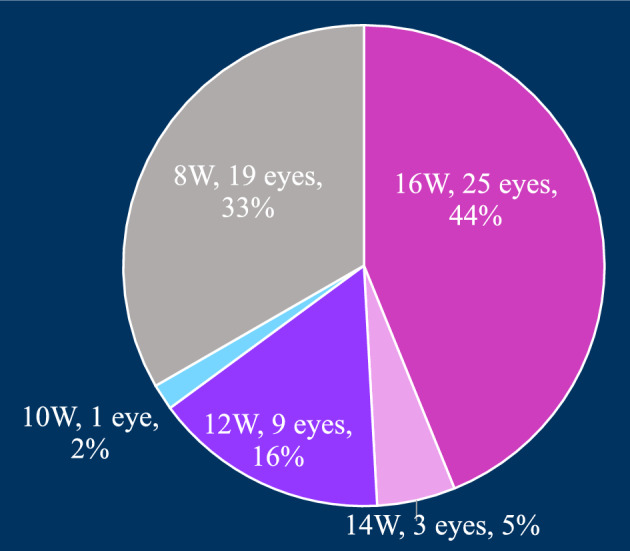
Proportions of patients with dry macula at 1 year after faricimab treatment, categorised by lesion type. A total of 37 out of 57 eyes (65%) achieved dry macula. *PCV* polypoidal choroidal vasculopathy.

The cumulative number of injections administered within the initial year was distributed as follows: six injections in 29 eyes (51%), seven injections in 13 eyes (23%), eight injections in 13 eyes (23%), and nine injections in two eyes (3%). The average number of injections per eye was 6.8 ± 0.9 (Fig. 6). Correspondingly, the distribution of patients based on treatment intervals at 1 year was as follows: 19 eyes (33%) at 8 weeks, one eye (2%) at 10 weeks, nine eyes (16%) at 12 weeks, three eyes (5%) at 14 weeks, and 25 eyes (44%) at 16 weeks (Fig. 7). Notably, all patients with a treatment interval of 16 weeks exhibited a dry macula. Conversely, among patients with an 8-week interval, 17 out of 19 eyes (89%) presented with exudative changes in the macula. At baseline, PED was identified in 34 (60%) out of 57 eyes. Over the course of 1 year, either complete disappearance or a reduction in PED was observed in 19 eyes (33%).

During the loading therapy, RPE tears were identified in two patients: an 83-year-old male with both type 1 and type 2 conditions, and a 77-year-old male diagnosed with PCV. Additionally, a 67-year-old female patient developed iritis after the sixth administration of faricimab; however, this iritis promptly resolved with the application of topical steroid eye drops.

**Figure 8:**
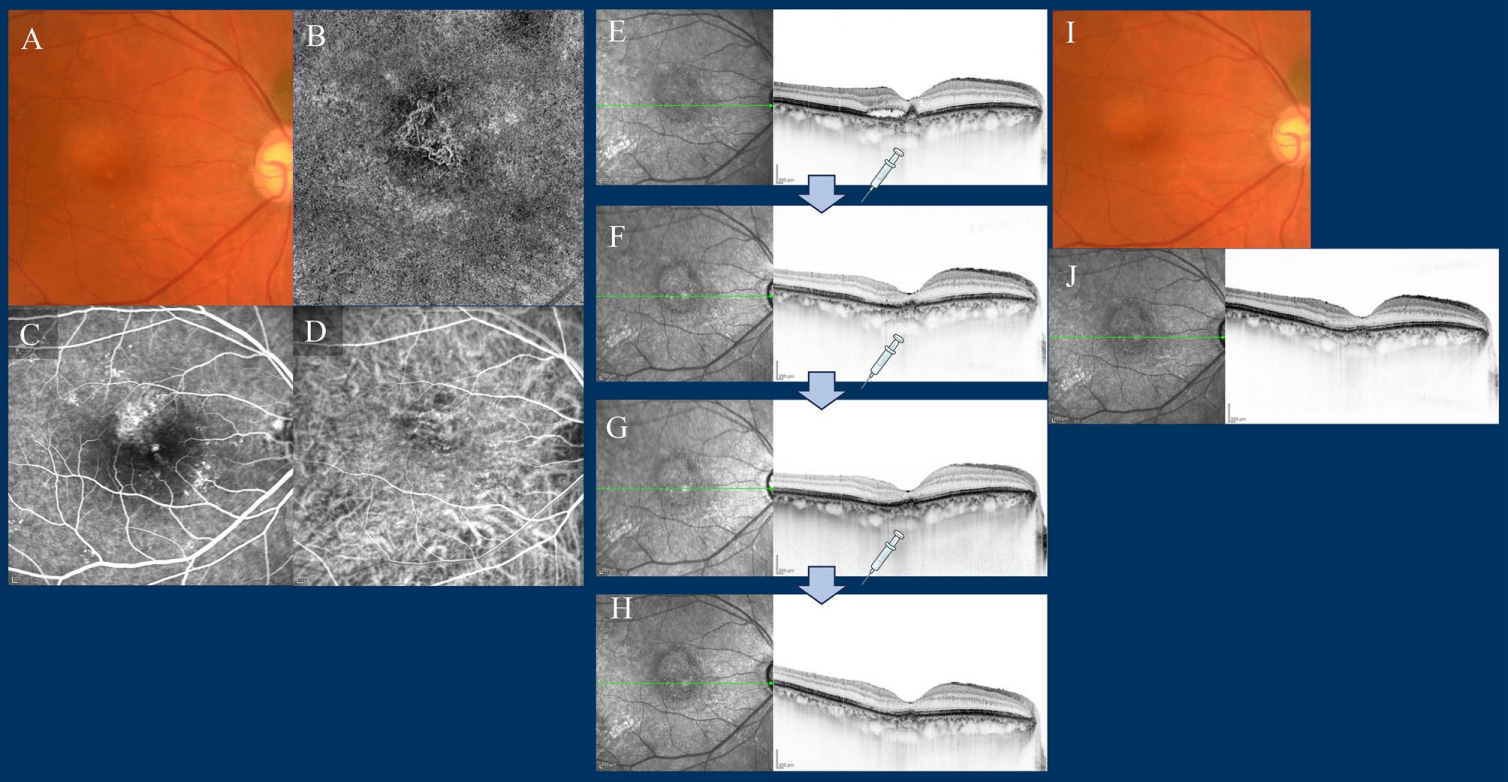
Case 1—A 70-year-old male with type 1 macular neovascularisation (MNV) at baseline (**A**–**D**). The best corrected Snellen visual acuity was 20/25. (**A**) Fundus photography (FP) revealed pigment epithelial detachment (PED) with subretinal fluid (SRF) at the fovea. (**B**) Optical coherence tomography angiography (OCTA) revealed MNV. (**C**) Late-phase fluorescein angiography revealed leakage from the MNV at the macula. (**D**) Middle-phase indocyanine green angiography identified vascular network at the fovea. (**E**–**H**) OCT images at baseline and at loading therapy with faricimab. At baseline, OCT revealed a small PED with SRF. The PED regressed during the loading therapy, and subretinal fluid was absorbed at 1, 2, and 3 months after the first injection of faricimab. (**I**,**J**) Fundus photography and OCT at 1 year; FP revealed no recurrence, and OCT showed no recurrence of fluid changes. At 15 months, six faricimab injections were administered, with treatment intervals extending up to a maximum of 16 weeks. His best corrected Snellen visual acuity was 20/16.

**Figure 9:**
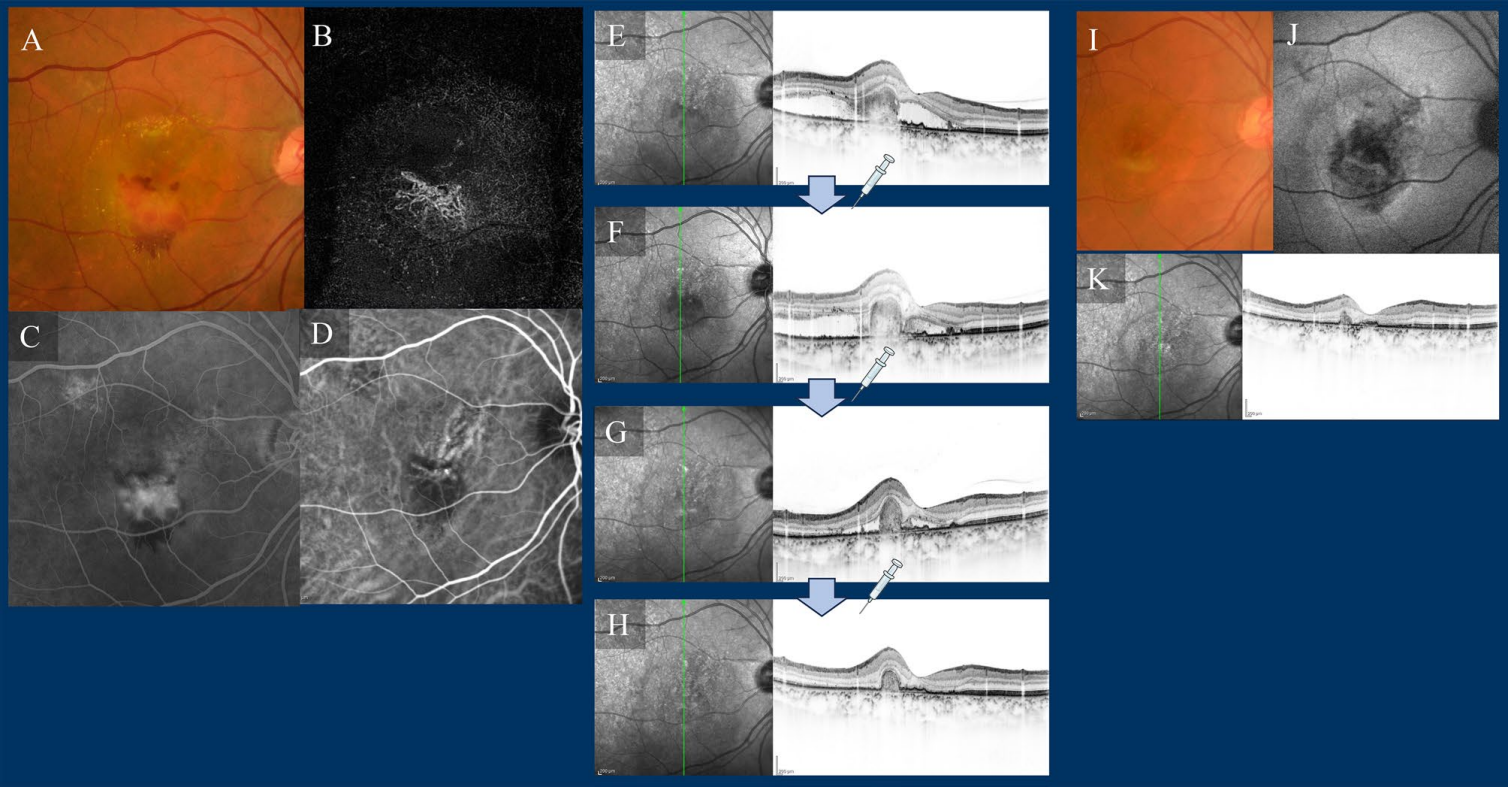
Case 2—A 59-year-old male with type 1 and type 2 macular neovascularisation (MNV). The best corrected Snellen visual acuity was 20/63 at baseline (**A**–**D**). (**A**) Fundus photography (FP) revealed a yellowish-grey bumped lesion at the fovea with subretinal fluid (SRF), subretinal haemorrhages, and hard exudate. (**B**) Optical coherence tomography angiography (OCTA) revealed MNV. (**C**) Late-phase fluorescein angiography detected a type 2 MNV. (**D**) Middle-phase indocyanine green angiography identified abnormal MNV at the fovea. (**E**–**H**) OCT at baseline and at loading therapy. (E) OCT revealed an SRF, accompanied by subretinal hyperreflective material, which corresponded to a yellowish-grey bumped lesion at the macula and SRF observed at 3 months. (**I**–**K**) *FP* fundus autofluorescence (FAF), and OCT at 1 year; FP showed no subretinal fluid (SRF) and a small scar lesion, FAF revealed a mild atrophic lesion around an original type 2 MNV, and OCT revealed no recurrence of fluid and a small bump lesion. At 15 months, the number of faricimab injections was six, with treatment intervals extending up to a maximum of 16 weeks. His best corrected Snellen visual acuity improved to 20/40.

**Figure 10:**
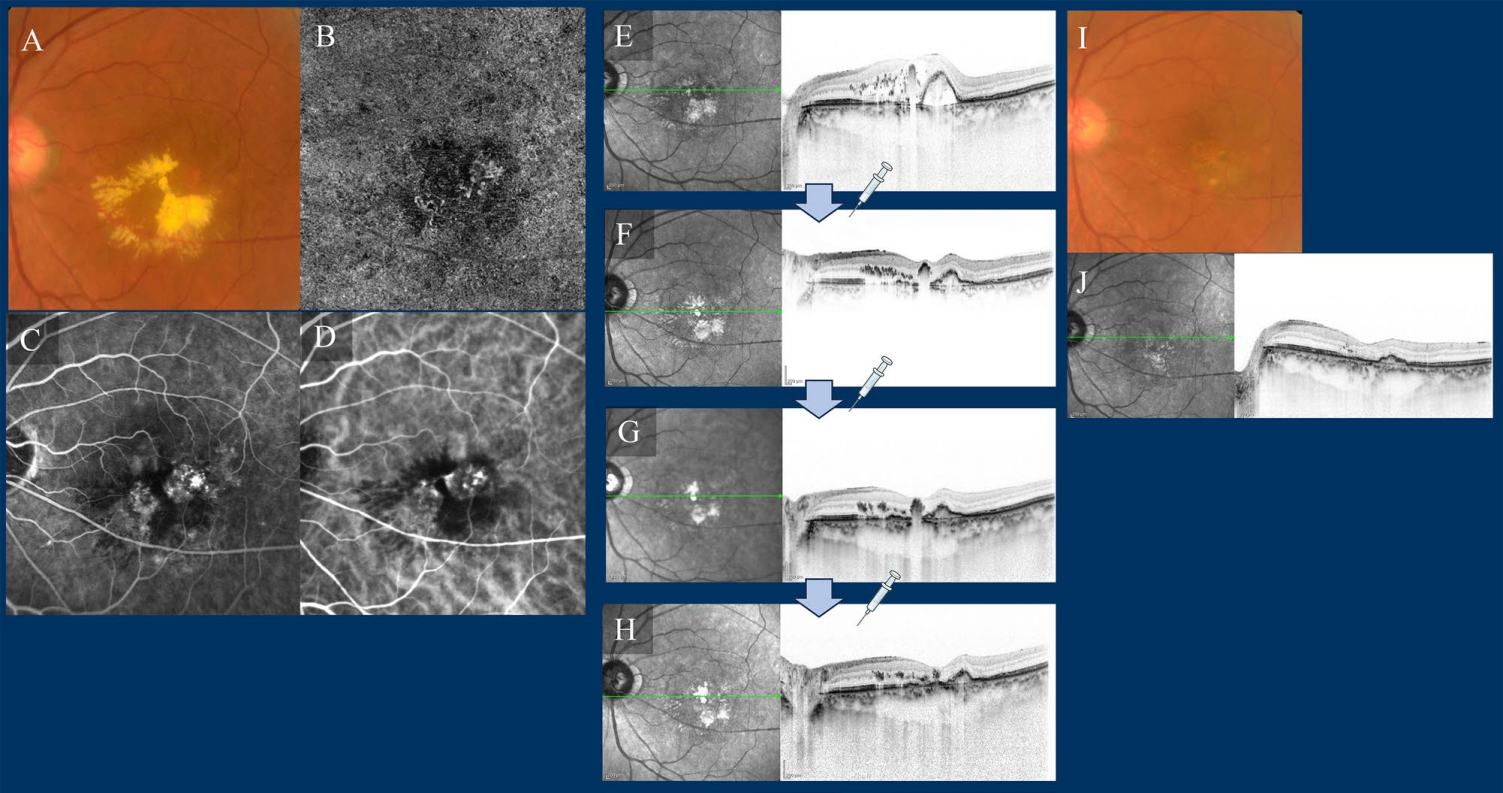
Case 3—A 74-year-old male with polypoidal choroidal vasculopathy (PCV) at baseline (**A**–**D**). The best corrected Snellen visual acuity was 20/320. (**A**) Fundus photography (FP) revealed an orange nodule surrounded by subretinal fluid with hard exudate at the fovea. (**B**) Optical coherence tomography angiography (OCTA) revealed MNV in the PED. (**C**) Late-phase fluorescein angiography detected granular leakage from the PED. (**D**) Middle-phase indocyanine green angiography identified polypoidal lesions at the fovea. (**E**–**H**) OCT images at baseline and at loading therapy. OCT revealed a sharp peaked pigment epithelial detachment (PED) accompanied by shallow subretinal fluid (SRF) with hard exudate at baseline; at 3 months, the SRF absorbed and the PED regressed. (**I**,**J**) FP and OCT at 1 year; FP showed no recurrence of fluid and that the hard exudate was almost absorbed, and OCT revealed no recurrence of fluid. At 15 months, the patient was administered six faricimab injections, with treatment intervals extending up to a maximum of 16 weeks. His best corrected Snellen visual acuity improved to 20/100.

**Figure 11:**
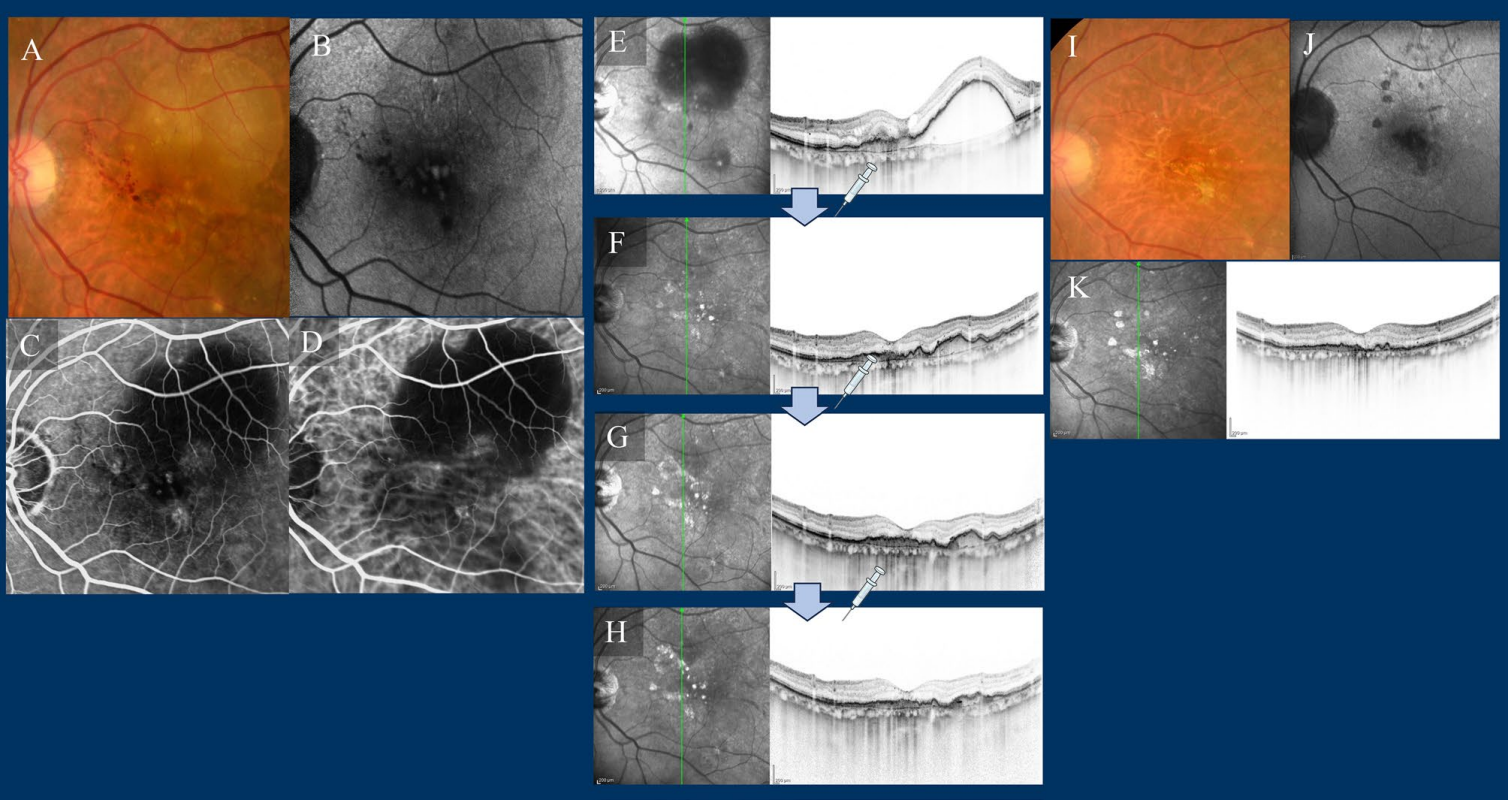
Case 4—A 90-year-old female with type 3 macular neovascularisation (MNV) at baseline (**A**–**D**). The best corrected Snellen visual acuity was 20/200. (**A**) Fundus photography (FP) revealed a pigment epithelial detachment (PED) with intraretinal fluid (IRF) and intraretinal haemorrhage at the fovea. (**B**) Fundus autofluorescence (FAF) revealed no macular atrophy. (**C**) Late-phase fluorescein angiography detected dye leakage from a type 3 MNV. (**D**) Middle-phase indocyanine green angiography also identified type 3 neovascularisation. (**E**–**H**) Optical coherence tomography (OCT) images at baseline and at loading therapy. OCT revealed a large PED with IRF at baseline and just 1 month after the first faricimab injection, the large pigment epithelial detachment (PED) visible at baseline rapidly regressed, and intraretinal fluid (IRF) had completely absorbed. (**I**–**K**) FP, FAF, and OCT at 1 year; FP showed no recurrence of the IRF, FAF revealed small patchy atrophy, and OCT revealed no recurrence of IRF or intraretinal haemorrhages. The patient was administered six faricimab injections, with treatment intervals extending up to a maximum of 16 weeks at 15 months. Her best corrected Snellen visual acuity improved to 20/100.

No other ocular or systemic complications, such as arterial thromboembolism, were observed throughout the study period. Comprehensive comparisons of demographic and clinical characteristics among patients with type 1 and/or type 2 MNV, PCV, and type 3 MNV are presented at baseline and 1-year post-commencement of faricimab treatment in Table 2, Supplementary Table S2, and Supplementary Figs. S1 and S2 online. Furthermore, representative cases treated with faricimab are visually depicted in Figs. 8, 9, 10 and 11.

**Table 2:** Comparison of demographic and clinical characteristics among cases with type 1 and/or type 2 macular neovascularisation (MNV), polypoidal choroidal vasculopathy (PCV), and type 3 MNV at baseline and at 1 year after initiation of faricimab treatment.

	Total	Type 1 and/or type 2 MNV	PCV	Type 3 MNV
Number of eyes, n (%)	57	30 (53%)	20 (35%)	7 (12%)
Female (%)	15 (27%)	7 (23%)	3 (15%)	5 (86%)
Mean age (years)	76 ± 10	76 ± 10	73 ± 9	85 ± 6
BCVA at baseline (logMAR)	0.44 ± 0.46	0.48 ± 0.50	0.30 ± 0.33	0.66 ± 0.52
BCVA at 1 year (logMAR) (the mean BCVA in logMAR at the final visit within the 12–15-month period)	0.34 ± 0.48	0.36 ± 0.50	0.16 ± 0.26	0.71 ± 0.57
BCVA at 12 months (logMAR): LOCF	0.34 ± 0.47	0.39 ± 0.51	0.14 ± 0.23	0.68 ± 0.58
BCVA at 15 months (logMAR): LOCF	0.35 ± 0.48	0.39 ± 0.52	0.15 ± 0.26	0.71 ± 0.57
Mean CFT at baseline (μm)	326 ± 149	347 ± 155	294 ± 102	328 ± 229
Mean CFT at 12 months (μm): LOCF	199 ± 84	213 ± 86	195 ± 81	153 ± 82
Mean CFT at 15 months (μm): LOCF	195 ± 82	210 ± 79	187 ± 86	155 ± 81
Mean SCT at baseline (μm)	220 ± 95	219 ± 103	242 ± 89	161 ± 48
Mean SCT at 12 months (μm): LOCF	199 ± 84	192 ± 93	223 ± 71	157 ± 59
Mean SCT at 15 months (μm): LOCF	195 ± 78	186 ± 84	223 ± 66	154 ± 59

In type 3 MNV, female dominance was 6 eyes of 5 female.

*BCVA* best-corrected visual acuity, *LOCF* last observation carried forward, *CFT* central foveal thickness, *SCT* subfoveal choroidal thickness.

## Discussion

The use of faricimab within a TAE regimen for treatment-naïve nAMD patients demonstrated significant improvements in both visual acuity and retinal morphology after 1 year. Notably, 44% of patients in this study successfully maintained a treatment interval of 16 weeks. Furthermore, faricimab led to a dry macula in 65% of all cases, remarkably in a relatively high proportion of patients presenting with type 3 MNV (86%).

The 1-year results of the TENAYA and LUCERNE international phase III clinical trials, which evaluated the efficacy of faricimab in patients with nAMD, demonstrated significant improvements in visual function^5^. Our study aligns with these findings, revealing a substantial improvement in visual acuity over a 1-year period, as evident in both raw data and when employing the LOCF method. Moreover, the improvements in retinal morphology observed in our study were consistent with those reported in these pivotal trials^5^. Our results consistently corroborate advancements in visual recovery and retinal morphology, as evidenced by the aforementioned clinical trials^5^.

Our study observed that 44% of patients maintained a 16-week treatment interval at the 1-year mark, closely mirroring the 45.3% reported in the combined TENAYA and LUCERNE analyses^5^. Notably, the ALTAIR study, which investigated aflibercept in a TAE regimen for up to 56 weeks, reported a similar proportion, with 40% of patients maintaining 16-week intervals^11^. These findings suggest that each anti-VEGF treatment might be effective for a subset of patients with nAMD, potentially enabling longer treatment intervals. Moreover, by examining shorter treatment intervals, the ALTAIR study indicated that approximately 30–40% of patients maintained 8-week intervals at 56 weeks, similar to the 33% observed in our study^11^. This similarity suggests that specific anti-VEGF therapies may not uniformly support extending the treatment intervals for a certain percentage of patients with nAMD. Taken together, these results suggest that while various anti-VEGF treatments effectively enable longer treatment intervals for a subset of nAMD patients, there is variability in the proportion of individuals achieving specific interval extensions. This variability suggests a lack of universal response among individuals to different anti-VEGF therapies. Further, our treatment regimen differed from that of the TENAYA and LUCERNE trials. In our study, the extension or shortening of treatment intervals was determined based on the presence of a dry macula, and no instances of haemorrhage occurred without accompanying vision loss. However, in those clinical trials, a fluid thickness of < 50 um was determined to be a dry macula. This difference may have led to the higher proportion of patients with treatment intervals of 12 or 16 weeks at 12 months, compared to the findings of this study.

In this study, we assessed the efficacy of faricimab in improving visual acuity among patients with nAMD, encompassing types 1 and 2 MNV and PCV. The observed effect was similar to the 1-year treatment outcomes reported in previous studies. Specifically, in cases of PCV, treatment responses regarding complete regression of polypoidal lesions varied across different anti-VEGF agents and photodynamic therapy. Ranibizumab showed a complete regression rate of 25%^12^, while in our previous study, aflibercept administered once every 2 months showed a rate of 50%^13,14^. Brolucizumab showed a rate of 79–90%^15,16^, and photodynamic therapy showed a rate of over 70%^17,18^. Our findings demonstrated a noteworthy 50% rate of complete regression following 1 year of faricimab treatment for PCV. Despite a reduction in PED detection from 11 out of 20 cases (55%) to four out of 20 cases (20%), a dry macula was achieved in 60% of patients 1 year after undergoing faricimab treatment for PCV. These findings underscore the potential efficacy of faricimab in promoting regression of PCV manifestations and highlight the importance of assessing multiple parameters, including PED and macular status, to comprehensively evaluate treatment outcomes.

At 15 months after the initial administration of faricimab for nAMD, including 35% of PCV cases, SCT decreased to 195 ± 82 μm, reflecting an 11% reduction from its baseline measurement. This reduction closely aligns with our previously reported findings at 3 months post-faricimab treatment, where a reduction of 12% (in nAMD, including 35% of PCV cases) was noted, from 215 ± 95 μm at baseline to 189 ± 82 μm at 3 months^6^. Remarkably, the ratio of SCT reduction remained consistent over 1 year. In comparison, our previous study reported a reduction from 268 ± 101 μm at baseline to 232.4 ± 99.6 μm at 1 year, with the use of aflibercept in the treatment of nAMD (including 59.7% of PCV cases). This corresponded to a reduction of 13.3% in SCT at the 1-year time point^19^. The trend depicting a reduction in SCT between faricimab and aflibercept treatments appeared to be parallel. In studies involving brolucizumab, the reported reductions at 1 year ranged between 14% (in PCV cases, reducing from 268 ± 101 μm at baseline to 232.4 ± 99.6 μm at 1 year)^20^ and 20% (in type 1 MNV, including 51.1% of PCV cases, reducing from 253 ± 91 at baseline to 206 ± 80 at 1 year)^21^, showcasing a potentially greater reduction compared to that achieved with faricimab. This discrepancy between faricimab and brolucizumab treatment might be correlated with the higher incidence of dry macula in PCV or nAMD patients following brolucizumab treatment^21^.

Regarding type 3 MNV, faricimab demonstrated substantial efficacy, resulting in a dry macula in 86% of cases while preserving visual acuity. Notably, our analysis included only seven eyes with type 3 MNV. Comparable studies often struggle to demonstrate significant visual acuity improvements in type 3 MNV due to relatively good baseline visual acuity and limited sample sizes for this specific subtype^22,23^.These findings underscore the potential of faricimab across various nAMD subtypes, aligning with or surpassing the outcomes observed with established treatments such as aflibercept, and offering promising implications for its clinical efficacy. However, further investigations with larger cohorts are warranted, especially in rarer subtypes such as type 3 MNV, to validate these findings and refine treatment paradigms.

Taking into account the findings of this study in conjunction with those of previous studies, comparable results were observed between faricimab and aflibercept treatment in terms of BCVA at 1 year post-treatment, improvement in retinal thickness, reduction in SCT, and regression of polyps^5,6,24^. Notably, a high-resolution ratio of retinal fluid in nAMD during the loading therapy phase was observed with faricimab treatment^6,24^. Moreover, the potential for inhibiting the expansion of macular atrophy^25^, prohibiting fibrosis^3,26^, along with the stability of choroidal vasculature attributable to protection against pericyte loss, was suggested with the use of faricimab over the long term. To thoroughly assess these effects, extended follow-up periods are imperative. This investigation highlights the need for a comprehensive understanding of the sustained benefits associated with faricimab in the management of nAMD, with emphasis on the prolonged observations to elucidate its impact, specifically in terms of macular atrophy expansion and prevention of fibrosis.

Over the course of 1 year, two cases of RPE tears occurred during the loading therapy phase, and one case of iritis emerged following the sixth administration of faricimab. No other serious adverse events, either general or ocular, were observed during the study period. Faricimab, characterised by a modified Fc portion, is believed to enhance drug clearance through the excretory system^22^. These modifications appear to contribute to the safety profile of faricimab by minimising ocular and general adverse events.

This study has certain limitations. Despite involving multiple institutions, the sample size was limited. In addition, the retrospective nature of this study introduces inherent constraints. Notably, five patients with an insufficient response to faricimab were excluded and switched to aflibercept. This exclusion might have led to an overestimation of the treatment effects of faricimab in this cohort.

In conclusion, our findings suggest that faricimab demonstrates promise in contributing to both functional and anatomical improvements in retinas affected by nAMD over a 1-year period when administered through a TAE regimen. However, larger prospective studies are needed to further validate these findings and address the limitations of this study.

### Supplementary Information

Supplementary Figure S1.

Supplementary Figure S2.

Supplementary Tables.

## Data Availability

The datasets generated and analysed in the current study are available from the corresponding author on reasonable request.
