# Supplementary material for: One-year outcomes and safety assessment of faricimab in treatment-naïve patients with neovascular age-related macular degeneration in Japan
Source: Sci Rep. 2024 May 22;14:11681. doi: 10.1038/s41598-024-62559-1 (PMC11111667; doi:10.1038/s41598-024-62559-1)
Supplement: Supplementary file 1 — Supplementary Figure S1. [file 41598_2024_62559_MOESM1_ESM.pdf]

Supplementary figure 1

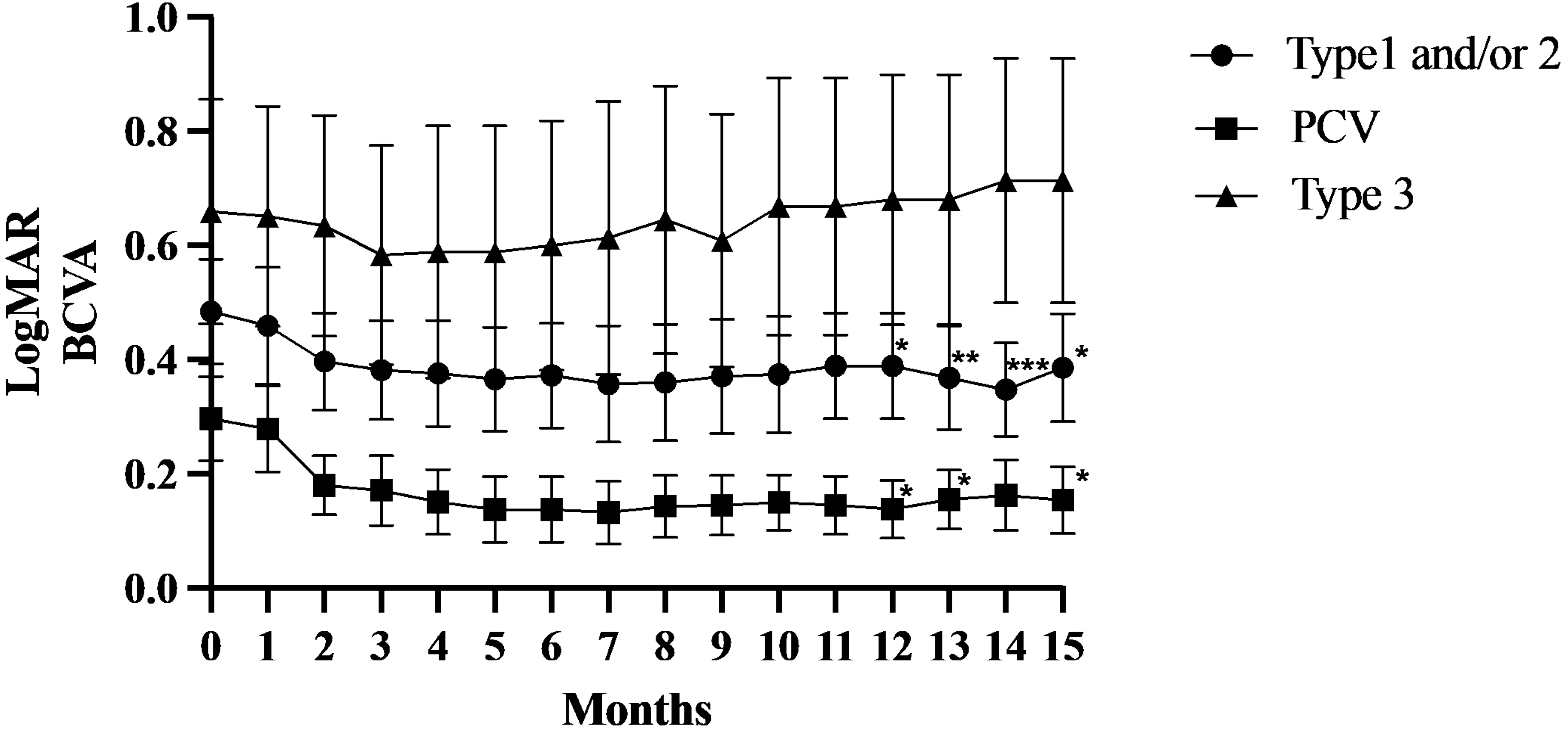

Supplementary Figure S1.
Changes in best-corrected visual acuity (BCVA) among all lesion types treated with faricimab for 1 year.
The data was analysed using last observation carried forward (LOCF) and shown as mean ± SE.
*: $p < 0.05$, **: $p < 0.01$, ***: $p < 0.001$. PCV: polypoidal choroidal vasculopathy.
