# Supplementary material for: One-year outcomes and safety assessment of faricimab in treatment-naïve patients with neovascular age-related macular degeneration in Japan
Source: Sci Rep. 2024 May 22;14:11681. doi: 10.1038/s41598-024-62559-1 (PMC11111667; doi:10.1038/s41598-024-62559-1)
Supplement: Supplementary file 2 — Supplementary Figure S2. [file 41598_2024_62559_MOESM2_ESM.pdf]

Supplementary figure 2

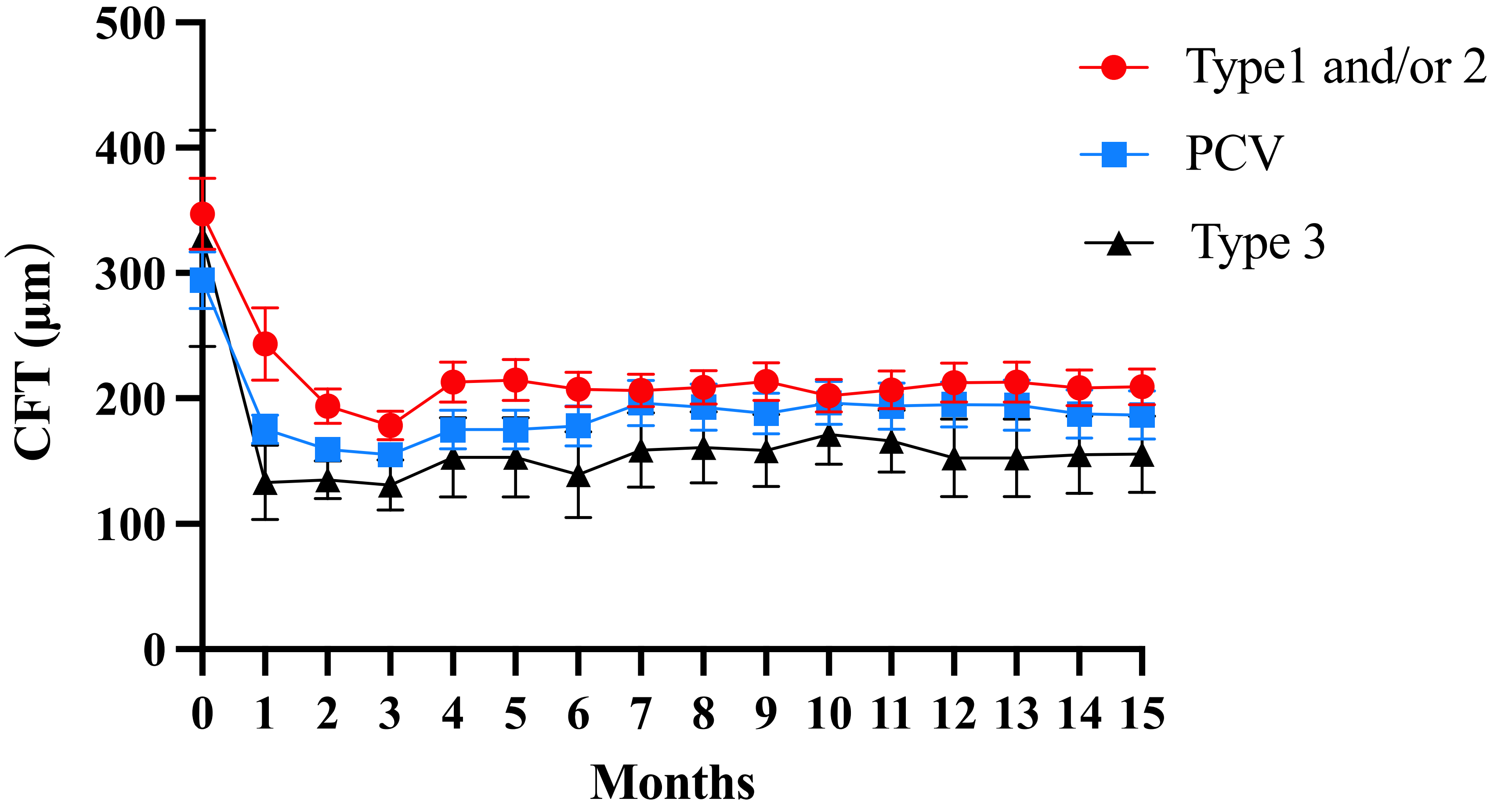


Supplementary Figure S2.
Changes in average central foveal thickness (CFT) among every lesion type treated with faricimab injections for 1 year. The data was analysed using last observation carried forward (LOCF), and shown as mean ± SE.
