# Supplementary material for: One-year outcomes and safety assessment of faricimab in treatment-naïve patients with neovascular age-related macular degeneration in Japan
Source: Sci Rep. 2024 May 22;14:11681. doi: 10.1038/s41598-024-62559-1 (PMC11111667; doi:10.1038/s41598-024-62559-1)
Supplement: Supplementary file 3 — Supplementary Tables. [file 41598_2024_62559_MOESM3_ESM.pdf]

Supplementary table 1

Analysis using a mixed-effects models for repeated measures (MMRM)

	P value
1.Visual acuity	
Pretreatment vs BCVA in logMAR at the final visit within the 12–15-month period	0.0006
2. CFT	
Pretreatment vs CFT at the final visit within the 12-15-month period	<0.0001
3. SCT	
Pretreatment vs SCT at the final visit within the 12-15-month period	0.0053

BCVA: best-corrected visual acuity, CFT: central foveal thickness, SCT: subfoveal choroidal thickness.

Supplementary table 2

Median (25%Pecentile-75%Percentile)	Total	Type 1 and/or Type 2 MNV	PCV	Type 3 MNV
BCVA at baseline (logMAR)	0.30 (0.10-0.70)	0.35 (0.10-0.70)	0.26 (0.02-0.49)	0.52 (0.22-1.16)
BCVA at 1 year (logMAR)<br>(the median BCVA in logMAR at the final visit within the 12–15-month period)	0.15 (0-0.52)	0.22 (0.03-0.70)	0.10 (-0.06-0.22)	0.82 (0.10-1.16)
BCVA at 12 months (logMAR): LOCF	0.15 (0-0.70)	0.22 (0-0.70)	0.10 (-0.06-0.22)	0.82 (0.10-1.10)
BCVA at 15 months (logMAR): LOCF	0.15(0-0.52)	0.22 (0.03-0.70)	0.10 (-0.06-0.22)	0.82 (0.10-1.16)
Median CFT at baseline (μm)	280 (228-384)	278 (245-439)	299(220-340)	245 (202-394)
Median CFT at 12 months (μm): LOCF	188 (148-226)	204 (154-223)	180(143-232)	147 (76-238)
Median CFT at 15 months (μm): LOCF	188 (135-226)	193 (159-229)	158(134-218)	129 (78-261)
Median SCT at baseline (μm)	215 (138-302)	216 (135-314)	244 (156-313)	138 (133-180)
Median SCT at 12 months (μm): LOCF	173 (138-259)	160 (132-255)	235 (147-294)	155 (119-175)
Median SCT at 15 months (μm): LOCF	178 (141-255)	160 (140-251)	217 (165-284)	144 (119-166)
In type 3 MNV, female dominance was 6 eyes of 5 female				

Comparison of demographic and clinical characteristics among cases with Type 1 and/or Type 2 macular neovascularisation (MNV), polypoidal choroidal vasculopathy (PCV), and Type 3 MNV at baseline and at 1 year after initiation of faricimab treatment. BCVA: best-corrected visual acuity, LOCF: last observation carried forward, CFT: central foveal thickness, SCT: subfoveal choroidal thickness.
